# Cost-Utility Analysis of Mirabegron Compared to Solifenacin in the Treatment of Overactive Bladder (OAB) in Iran

**DOI:** 10.5812/ijpr-136447

**Published:** 2023-11-08

**Authors:** Zahra Karimi Majd, Ghader Mohammadnezhad, Saeed Taheri, Nazila Yousefi

**Affiliations:** 1 Department of Pharmacoeconomics and Pharma Management, School of Pharmacy, Shahid Beheshti University of Medical Sciences, Tehran, Iran; 2 School of Pharmacy, Shahid Beheshti University of Medical Sciences, Tehran, Iran

**Keywords:** Mirabegron, Cost-Utility, Pharmacoeconomics, Overactive Bladder, Economic Evaluation, Urology

## Abstract

**Background:**

Overactive bladder (OAB) is a symptomatic condition characterized by urinary urgency with or without incontinence, usually associated with frequent daytime urination, enuresis, and nocturia.

**Objectives:**

This economic evaluation was aimed at assessing the cost-effectiveness of mirabegron versus solifenacin in the treatment of OAB patients from a payer’s perspective in Iran.

**Methods:**

A Markov model with a 5-year time horizon was used. The model consisted of five health states, and OAB patients with an average age of 60 years entered the cycle from the persistent state. Transition probabilities were based on published trials, clinical judgments, and expert opinions. Resource use and costs, including those for medications and adverse events, were extracted from the literature and tariff book, and all costs are presented in 2019 US dollars with a 5% discount rate for the costs and utilities. The incremental cost-effectiveness ratio (ICER) and quality-adjusted life-years (QALYs) were computed for medications, and sensitivity analyses were used to test the robustness of the results.

**Results:**

Average per-patient treatment costs were $24,720.7 and $24,668.6 for mirabegron and solifenacin, respectively. Mirabegron was expected to produce higher QALYs than solifenacin (3.20 vs. 3.19). Mirabegron had an ICER of $531.3 over solifenacin, lower than the willingness-to-pay (WTP) threshold. The probabilistic analysis showed mirabegron cost-effectiveness in 80% of simulations at the WTP of $2709/QALY.

**Conclusions:**

Compared to solifenacin, mirabegron was more cost-effective in OAB patients in the Iranian healthcare system.

## 1. Background

Overactive bladder (OAB) is a symptomatic condition characterized by urinary urgency with or without incontinence, usually associated with frequent daytime urination, enuresis, and nocturia. This condition occurs without urinary tract infection (UTI) or other pathological conditions (1, 2). This syndrome occurs in both genders and is more prevalent in the elderly. The overall prevalence of OAB was 20.1% worldwide in 2018, increasing from 455 to 546 million individuals, calculated within 2008-2018. The prevalence of OAB in epidemiological studies varies from 7% to 27% and 9% to 43% in men and women, respectively (3-5). In a 2009 epidemiological study in Iran, the prevalence of OAB in women aged 15 - 55 years was 18.2% (6).

Overactive bladder symptoms can interfere with daily activities, sleep, mental health, and personal relationships. In addition, OAB symptoms negatively impact health-related quality of life (HRQoL), and the evidence suggests that comorbidities, such as depression, bone fractures, skin infections, and UTIs, might be directly related to it (1). For the initial treatment of OAB in Iran, conservative management (e.g., bladder training and lifestyle modification), followed by primary pharmacotherapy, botulinum toxin (BTX), or surgery (e.g., sacral nerve stimulation), is currently recommended (7, 8).

Antimuscarinics (e.g., oxybutynin, tolterodine, and solifenacin) have been the mainstay of the first-line treatment for OAB patients, and mirabegron has not yet been added to the Iran drug list (IDL) (9, 10). With the non-selective feature, antimuscarinics have an affinity with all muscarinic receptors and cause side effects, such as constipation, xerostomia, dry eye syndrome, and blurred vision, that might affect patient compliance (11, 12). The first-in-class oral beta3-adrenergic agonist, mirabegron, with comparable efficacy to antimuscarinics, a lower xerostomia incidence, and an enhanced tolerability profile, is just about to enter the IDL (13-15). Although it has not yet entered the Iranian pharmaceutical market, due to its numerous benefits, many physicians and patients are inclined to add it to the treatment of this disease. However, new treatments are usually associated with higher costs, and any new drug must demonstrate its value vis-a-vis its alternatives.

## 2. Objectives

This study aimed to evaluate the cost-effectiveness of mirabegron compared to solifenacin for the treatment of OAB from the payer’s perspective in the Iranian healthcare system.

## 3. Methods

### 3.1. Model Overview and Outcomes

A Markov model was developed to analyze the cost-utility effect of mirabegron 50 mg/d, compared to antimuscarinic treatment, for OAB in Iran in 2019. After consultation with an expert panel of urologists and observation of clinical practice in Iran, solifenacin 5 and/or 10 mg was selected as the comparison arm in the first-line of treatment and tolterodine 2 and/or 4 mg as the second-line. The data from randomized controlled trials (SYNERGY II) and meta-analysis of international studies were used to obtain efficacy and clinical safety parameters (16-19).

In this study, a hypothetical cohort of 1000 OAB patients with a mean age of 60 years and a male-to-female ratio of 1:5 was used as the target population based on epidemiological statistics (20). The model simulated treatment options, disease state, comorbidities, and their impact on costs and health outcomes. The study was designed and conducted from the payer’s perspective. The direct costs considered in the model included medication costs, medical services, OAB comorbidities, and the cost of hospital services. The clinical efficacy of mirabegron was stated using factors including reduction in urinary urgency, micturition, enuresis, nocturia, urinary urgency episodes, and incontinence. Finally, these factors were reported as primary clinical outcomes in terms of quality-adjusted life-years (QALYs) (21).

### 3.2. Model Structure

A Markov model consisting of four states and death was considered (22). The patient can switch between these states in monthly cycles. All patients entered the model from the persistence state and were assigned to treatment with mirabegron 50 mg or solifenacin 5 or 10 mg once daily as the first-line treatment. Below is a brief description of each disease state:

(1) Persistence: The patient is taking the appropriate medication and is stable.

(2) Non-persistence switching: A state in which the treatment regimen is changed due to lack of response or intolerance of first-line treatment.

(3) Non-persistence surgery: A state in which non-persistent patients are operated on at the discretion of the physician or due to non-persistence or lack of response to first- and second-line treatments.

(4) Non-persistence: When the patient discontinues medication for any reason.

(5) Death: The absorbing state of the model.

**Figure 1.**
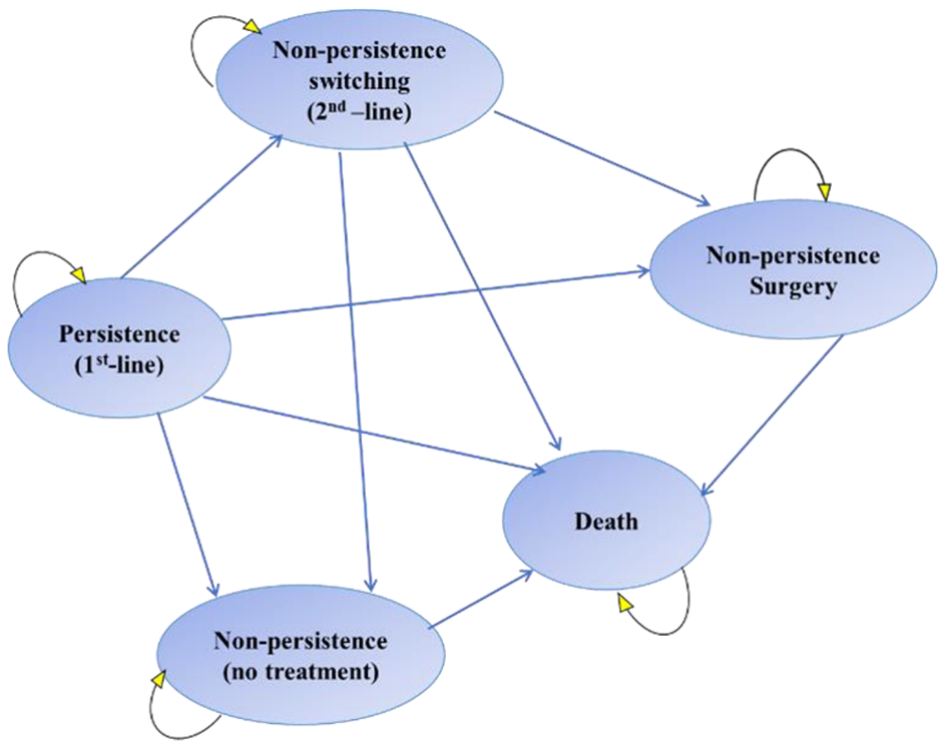
Model structure

After each monthly cycle, patients’ states either transitioned to a lower severity state, remained at the same severity level or worsened. The patients experienced OAB-related comorbidities and required incontinence pads and other interventions depending on severity. In addition, patients in the solifenacin arm suffered from the cognitive burden associated with antimuscarinics, which affected their benefit. Figure 1 shows a schematic structure of the Markov model used in this study (23, 24). Markov flowchart was programmed in TreeAge Pro Healthcare.

### 3.3. Model Input Parameters

The study has two main dimensions: The costs and clinical outcomes of the drugs being compared. The costs were taken from the 2019 national tariff book and the official price list. Only direct medical costs were considered and expressed in US dollars (USD) (based on the 2018 conversion rate: 42000 IRR), and clinical efficacy outcomes were expressed as QALY. Finally, the incremental cost-effectiveness ratio (ICER) described the relative incremental cost per additional QALY gained for mirabegron versus solifenacin.

Patient utility in each state was calculated as a function of incontinence and micturition. Tables 1 and 2 show that utility values were derived from the Overactive Bladder Questionnaire (OAB-q) and HRQoL according to symptom severity and then calculated using the matrix in Table 3 (25, 26). Additionally, Table 4 shows the calculated utility values derived from the European Quality of Life Five-Dimension Questionnaire (EQ-5D) and OAB-q index scores for each symptom severity level. There is no access to utility data in Iran, and the clinical data, including transition probabilities between different states, were extracted from previous equivalent studies using severity scores for each symptom obtained from a multinomial logistic regression model estimated from the SCORPIO trial (Table 1) (27, 28).

**Table 1.** Symptom Severity Levels: Definitions and Distribution of Patients at Baseline

Symptom Severity	Micturition		Incontinence	
	Mean No. of Micturition/day	Proportion of Patients, %	Mean No. of Incontinence Episodes/day	Proportion of Patients, %
**Level 1**	≤ 8	6.30	0	38.87
**Level 2**	> 8 to ≤ 10	30.69	1	18.84
**Level 3**	> 10 to ≤ 12	27.18	2	14.64
**Level 4**	> 12 to ≤ 14	19.46	3	9.18
**Level 5**	> 14	16.37	> 3	18.47

**Table 2.** Transition Probabilities Between Symptom Severity Levels for Mirabegron 50 mg and Solifenacin 10 mg

	Mirabegron 50 mg						Solifenacin 10 mg				
**To:**	1	2		3	4	5	1	2	3	4	5
**From:**	**Severity Level at 3 Months**										
**Micturition frequency**											
1	0.760	0.215		0.020	0.003	0.001	0.737	0.235	0.024	0.004	0.001
2	0.335	0.484		0.158	0.019	0.004	0.305	0.496	0.174	0.021	0.005
3	0.110	0.336		0.400	0.108	0.046	0.095	0.327	0.418	0.115	0.046
4	0.032	0.149		0.364	0.273	0.183	0.027	0.141	0.371	0.281	0.180
5	0.014	0.044		0.125	0.214	0.602	0.005	0.024	0.103	0.238	0.629
**From:**	**Severity Level at 3 Months**										
**Incontinence**											
1	0.873	0.103		0.012	0.006	0.006	0.858	0.114	0.014	0.007	0.007
2	0.504	0.36		0.080	0.028	0.021	0.471	0.385	0.088	0.032	0.024
3	0.331	0.349		0.184	0.093	0.043	0.300	0.354	0.197	0.102	0.046
4	0.191	0.274		0.210	0.185	0.139	0.168	0.271	0.218	0.198	0.145
5	0.106	0.121		0.123	0.160	0.490	0.065	0.088	0.117	0.187	0.544

**Table 3.** Monthly Transition Probabilities

Input	Model Input	Calculation	Monthly Transition Probabilities ^a^
**Persistence ** ^**a**^			
Mirabegron 50 mg	31.7%	1-EXP(-(-LN(1-(1-31.7%)))/12)	0.091
Solifenacin 5/10 mg	22.0%	1-EXP(-(-LN(1-(1-22.0%)))/12)	0.119
Tolterodine ER 4 mg	19.7%	1-EXP(-(-LN(1-(1-19.7%)))/12)	0.127
Tolterodine IR 2/4 mg	19.7%	1-EXP(-(-LN(1-(1-19.7%)))/12)	0.127
Non-persistence switch to active treatment proportion	70%		
Non-persistence switch to no treatment proportion	30%		
Mortality	0.49%	1-EXP(-((0.49%/12)*1))	0.00041
Minimally invasive procedure	0.01%	-	0.0001
Depression	18.8%	1-EXP(-(-LN(1-(18.8%))/6))	0.03419302
Urinary tract infection	30.7%	1-EXP(-(-LN(1-(30.7%))/6))	0.05929048

Abbreviations: ER, extended-release; IR, immediate release.

^a^ The monthly transition probabilities for persistence are the probabilities of non-persistence on treatment. This is the proportion of the cohort at each cycle that discontinues treatment and transitions from the persistent health state to the non-persistence health state.

**Table 4.** Utility Values Derived from European Quality of Life Five-Dimension Questionnaire and Overactive Bladder Questionnaire Index Scores for Each Symptom Severity Level

Questionnaire and Incontinence Severity Level	Micturition Severity Level				
	1	2	3	4	5
**EQ-5D**					
1	0.85	0.83	0.81	0.80	0.79
2	0.83	0.81	0.79	0.78	0.77
3	0.82	0.80	0.78	0.77	0.76
4	0.80	0.78	0.76	0.75	0.74
5	0.79	0.77	0.75	0.74	0.73
**OAB-q**					
1	0.92	0.88	0.85	0.84	0.82
2	0.89	0.85	0.83	0.81	0.79
3	0.87	0.83	0.80	0.78	0.77
4	0.85	0.81	0.79	0.77	0.75
5	0.84	0.80	0.78	0.76	0.74

Abbreviations: EQ-5D, European Quality of Life Five-Dimension Questionnaire; OAB-q, Overactive Bladder Questionnaire.

Because this study was conducted from the payer’s perspective, only direct medical costs were included in the data analysis, such as the costs of medications (mirabegron 50 mg once daily, solifenacin 5 and/or 10 mg once daily, and tolterodine 2 mg twice daily), physician visits (every 3 months), hospitalization, surgery, follow-up, rehabilitation, treatment of side effects (including the cognitive burden of antimuscarinics), disease comorbidities, and urinary incontinence pad costs (Table 5). Based on the literature, it was estimated that 70% of non-persistent patients changed their treatment, and 30% entered the no-treatment state (24, 29). All the patients included in the model received the first-line treatment. Only 1% of the patients who did not respond to first- and/or second-line treatments were selected for minor surgery (20, 23).

**Table 5.** Costs of Drugs and Interventions

Cost Category and Product/Service	Unit Price ($)	Administration	Cost Per Each Cycle ($)
**Medication costs**			
Mirabegron	0.857 (per each 50 mg tablet)	Once daily	26.13
Solifenacin	0.259 (mean per each 5 and 10 mg tablet)	Once daily	7.89
Tolterodine	0.09 (mean per each 2 mg tablet)	Twice daily	5.49
**Medical services**			
Urology specialist visit	5.89	Every 3 months	1.96
Botulinum toxin A 100 IU	262.57	Per injection	-
Sacral neuromodulation	71.69	Per procedure	-
Bladder augmentation surgery	376.16	Per procedure	-
Pads used	1.73	2.5 pads/day for 10% of on-treatment patients; 5.5 pads/day for 50% of off-treatment patients	-
Cognitive burden	5.95	Monthly for patients on solifenacin	5.95
**Hospital service**			
Hospital service costs	11,785.51	Bed/day for the total costs	-

Given the nature of OAB and previous studies, the model’s time horizon was set at 5 years (30). A discount rate of 5.8% was considered for costs, as proposed by Abdoli in Iran. Discounting for utility values was 5%, the highest recommended rate globally, due to the galloping rate of inflation in Iran and based on the rate reported by Abdoli’s (31) studies to reduce the gap between the two discount rates (31, 32).

### 3.4. Model Outputs

The primary outcome was the ICER as cost per QALY gained. The willingness-to-pay (WTP) threshold was $2709/QALY, which is 1 × Iran’s gross domestic product (GDP)/per capita.

### 3.5. Sensitivity Analyses

Due to natural differences in populations and heterogeneity in external data collection, uncertainties in economic evaluation studies cannot be avoided. Therefore, to evaluate the robustness of the model, deterministic sensitivity analysis (DSA), including a two-way sensitivity analysis, was used to evaluate the effect of each of the input parameters in the ±20% range of the economic model on the final results and plotted in a tornado diagram. Then, a one-way sensitivity analysis was used to calculate and plot the effect of essential and influential parameters from the tornado diagram on the results in each case. Finally, a probabilistic sensitivity analysis (PSA) was performed. The Monte Carlo simulation method was used for the PSA, considering patients’ direct medical costs and discounted QALY rates in the treatment regimens of mirabegron and solifenacin in a cohort of 1,000 patients. The costs, transition probabilities, and utilities were entered into the model in a distributional form for the PSA.

## 4. Results

According to patient disposition results, after 12 months of treatment, more patients were in the persistence state with mirabegron than solifenacin (35% vs. 20%, respectively), which indicated that more patients adhered to mirabegron as the first-line treatment.

The calculated 5-year utility per patient was 3.20 and 3.19 QALYs for mirabegron and solifenacin, respectively. The 5-year cost per subject was $2,472.07 and $2,466.86 for mirabegron and solifenacin, respectively (Table 6). A WTP threshold of $2709/QALY gained was used to interpret the ICER in this study, as this is the maximum threshold used to determine the likelihood that treatment is cost-effective in Iran. The ICER calculated in this study was $531.31/QALY, which remained below the generally accepted threshold for WTP, implying that mirabegron is more expensive and effective.

**Table 6.** Cost-Utility Strategies

Strategy	Cost ($)	Effectiveness (QALYs)	Incremental Cost ($)	Incremental Effectiveness (QALYs)	ICER
**Mirabegron**	2,472.07	3.20	5.21	0.01	531.31
**Solifenacin**	2,466.86	3.19	-	-	-

Abbreviations: QALYs, quality-adjusted life-years; ICER, the incremental cost-effectiveness ratio.

**Figure 2.**
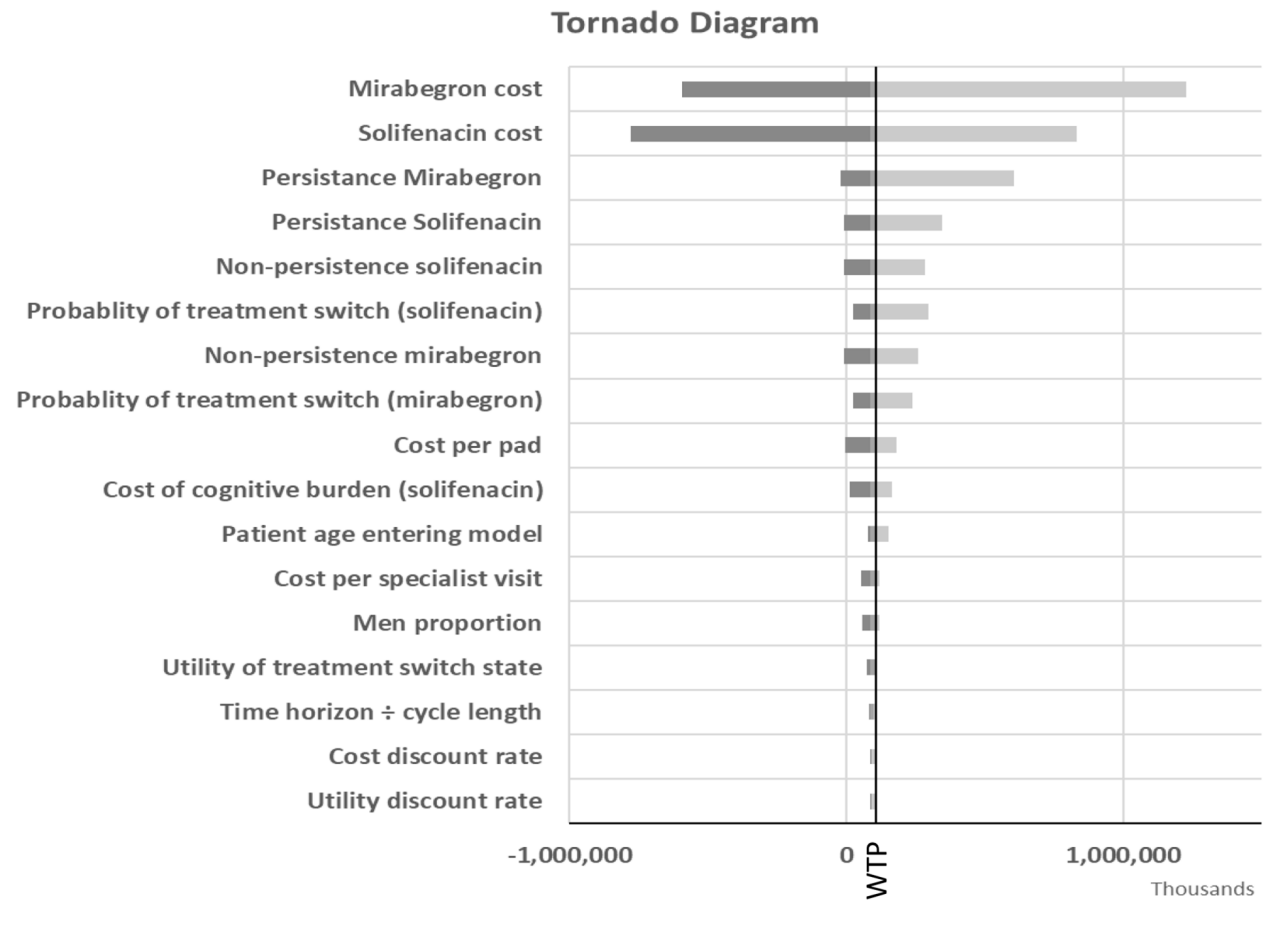
Deterministic sensitivity analysis

Figure 2 is the tornado diagram indicating the most influential model parameters in the ICER when comparing mirabegron to solifenacin. The results are shown with QALYs as the outcome measure. The model was most sensitive to the cost of mirabegron and solifenacin, persistence and non-persistence rates with each treatment option, and the probability of treatment switching. In all analyses, mirabegron remained cost-effective at the $531.31/QALY threshold.

**Figure 3.**
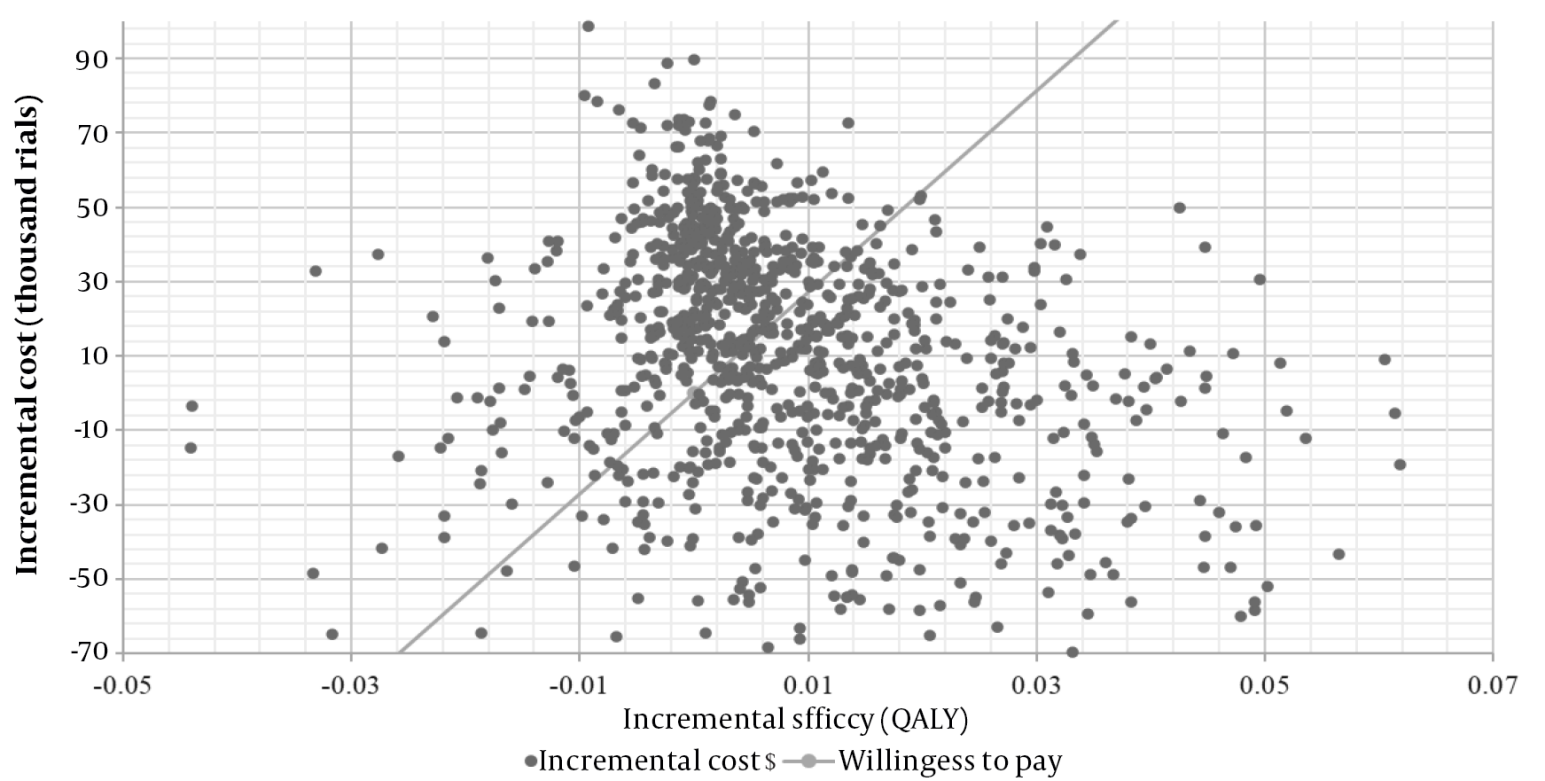
Incremental cost-effectiveness scatter plot

The PSA estimated the distribution of Monte Carlo simulation points for patients receiving mirabegron as the first-line treatment, compared to solifenacin, at the verge of the WTP of $2709 per QALY. This is presented in the corresponding cost-effectiveness acceptability curve (CEAC) (Figure 3). As shown in Figure 3, 81% of the mirabegron cases were in the cost-effective range; therefore, mirabegron was considered the cost-effective strategy.

## 5. Discussion

**Figure 4.**
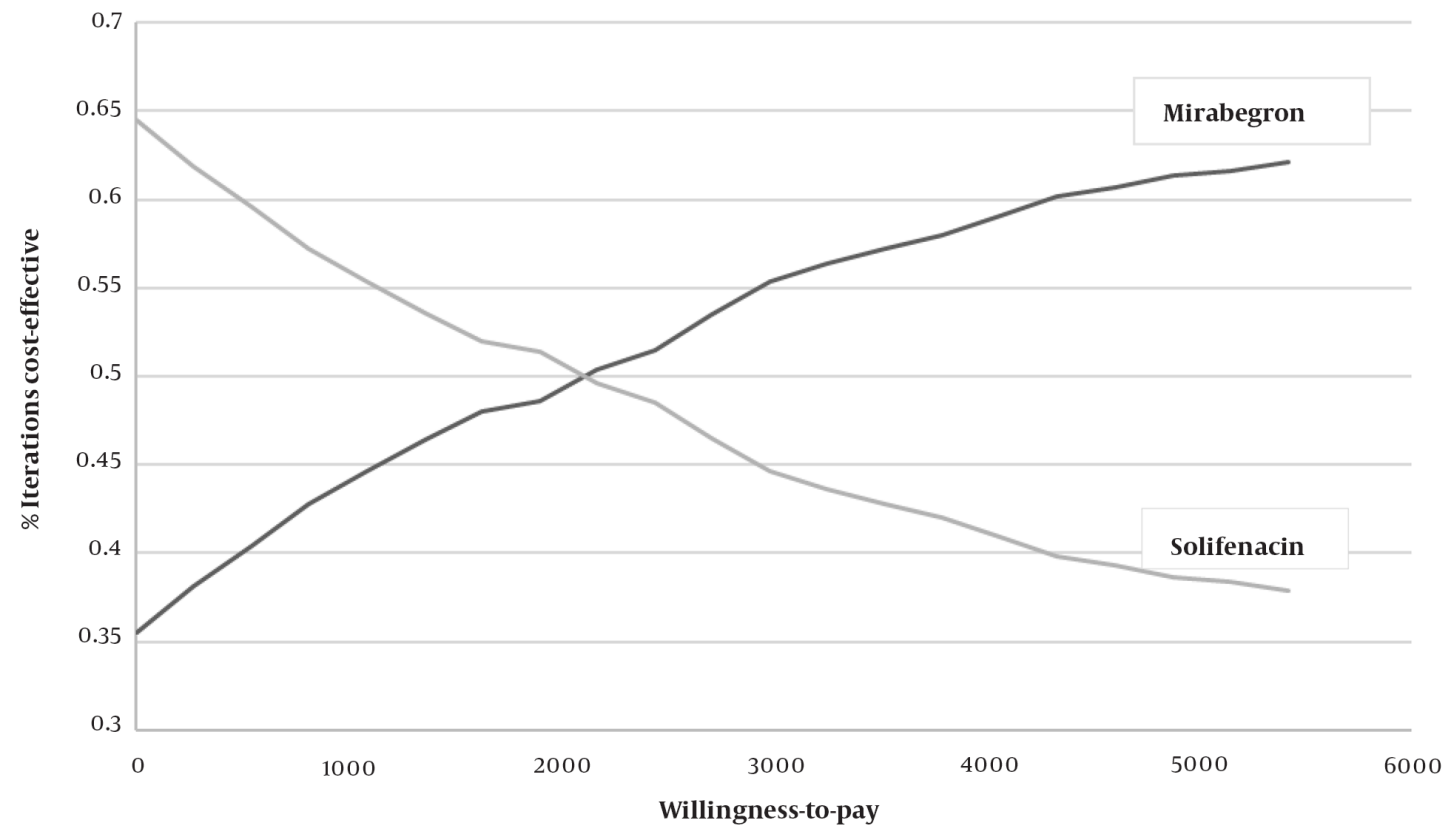
Probabilistic sensitivity analysis (PSA) results; cost-effectiveness acceptability curve (CEAC), mirabegron vs. solifenacin

This analytical cost-utility study was the first economic evaluation of mirabegron in Iran, a valuable study for healthcare decision-makers. According to the results, although mirabegron treatment was generally more expensive, these costs were offset by its greater effectiveness and adverse effects profile. The higher costs of the mirabegron strategy can be attributed to the fact that more patients stay in the persistent state, which uses more resources from the healthcare system. This study suggested that patients treated with mirabegron are more likely to adhere to the first-line treatment, resulting in more patients with controlled symptoms (Figure 4).

Recent real-world studies showed that patients treated with mirabegron as the first-line treatment have a higher persistence rate than other first-line therapies (24, 33). High persistence rates improve the patient’s quality of life and daily function by better controlling the symptoms. Due to the adverse effects of antimuscarinics, patients are more likely not to take these agents in the long term (30, 34, 35). Despite the higher cost of acquiring the drug, adherence to the treatment with mirabegron is higher, and follow-up costs are lower because fewer adverse events, particularly cognitive impairment, occur. This finding is in line with a recent systematic review and network meta-analysis by Nazir et al. (32) in 2014, which also shows that mirabegron has the same rate of dry mouth in patients compared to placebo but has a lower incidence than antimuscarinics (12, 36). It should be noted that the discontinuation of treatment can have various causes, and this issue should be considered in tailoring OAB treatment to the patient’s individual condition.

In a similar study in a developing country by Parise et al. (30), mirabegron was compared to oxybutynin extended-release (ER) and tolterodine ER. In this analysis in Colombia, mirabegron was a cost-effective option, assuming a WTP of 3 × GDP/per capita in Colombia (124.9 million Colombian pesos). Deterministic sensitivity analysis was most sensitive to the short- and long-term persistence of mirabegron and oxybutynin and utility losses associated with xerostomia. Probabilistic sensitivity analysis showed that in 99.5% and 100% of cohort simulations, mirabegron was cost-effective compared to oxybutynin and tolterodine ER. In another 2016 study in Russia (37), mirabegron treatment was 16% less expensive than solifenacin and 61% cost-saving as the second-line therapy than BTX over a one-year horizon. In another study conducted by Wielage et al. (as cited by Mohammadnezhad et al.), the cost-effectiveness of mirabegron in treating OAB from a US commercial health plan and medicare advantage perspective was evaluated over a 3-year time horizon. The analysis estimated that mirabegron is a cost-effective treatment for OAB from both perspectives due to fewer projected adverse events and comorbidities and better persistence (38).

This study has several strengths, including the inclusion of the costs and outcomes of each treatment strategy with and without side effects, the recruitment of the data from the SCORPIO trial as the most appropriate clinical trial on this topic in a five-year time horizon, and the use of tools consisting of the two validated EQ-5D and OAB-q instruments. However, the current study had some limitations that affected the results, which were not including a different dosage of mirabegron (25 mg). Although the present comparative strategy was a common first-line indication of OAB, various antimuscarinics and third-line therapies (including BTX, sacral nerve stimulation, and percutaneous tibial nerve stimulation) were not adopted as the comparison arm. Another limitation is that not all antimuscarinic side effects were included in the study, in the case of which the results would be more in favor of mirabegron.

### 5.1. Future Perspectives

It is expected that therapists and OAB patients will change their attitudes toward the use of beta-3 agonist drugs in the coming years. Antimuscarinic drugs, which currently have a significant market share in OAB disorder, are not considered a good choice as the first line of treatment for OAB due to their many side effects and consequent reduction in patient adherence to medication. Beta-3 agonists are a group of new and developing drugs for OAB that are as effective as antimuscarinics but have far fewer side effects. Beta-3 agonist drugs, such as mirabegron, along with fewer side effects and better acceptance by patients, lead to reduced costs associated with patient’s health and can have a higher market share in the OAB market in the future.

### 5.2. Conclusions

This comparative cost-utility analysis considered all predicted parameters affecting direct costs and utilities. This model suggests that OAB patients treated with mirabegron are more likely to stay on treatment and have a better adherence rate than the solifenacin group, which means better efficacy.
